# Physical Impairment, Insurance Coverage, and Healthcare Utilization Among U.S. Chinese Older Adults

**DOI:** 10.1093/geroni/igaa057.264

**Published:** 2020-12-16

**Authors:** Jonas Attilus, Mengting Li, Qun Le, XinQi Dong

**Affiliations:** 1 Rutgers University, New Brunswick, New Jersey, United States; 2 Rutgers University, New Brunswick,, New Jersey, United States

## Abstract

The relationship between physical impairment and healthcare utilization is well studied. However, few studies examined this relationship among immigrant older adults whose health insurance status may represent a barrier to healthcare use. This study aims to examine the relationship between physical impairment, health insurance, and types of healthcare utilization. The PINE Study provided data of 3,157 Chinese older adults age 60 and over. Most (70.67%) of them had insurance. Physical function was assessed by Activities of Daily Living and Instrumental Activities of Daily Living. Healthcare utilization was evaluated by the times of physician visit (PV), ER, and hospitalization, separately, in the past two years. Logistic regression was used. After adjusting for covariates, among the insured patients, every one unit increase in ADL impairment was associated with higher odds of ER visit (OR:1.32 [95%CI 1.21-1.45]) and hospitalization (OR: 1.37, [95%CI 1.25-1.50]). Every one greater IADL impairment was associated with higher odds of PV (OR: 1.26, [95%CI 1.12-1.43]), ER visit (OR: 1.21, [95%CI 1.16-1.26]), and hospitalization (OR: 1.23, [95%CI 1.18-1.28]). Among the non-insured, every one unit increase in ADL impairment was associated with higher odds of ER visit (OR: 1.82, [95%CI 1.19-2.78]) and hospitalization (OR: 3.05, [95%CI 1.51-6.16]). Every one unit increase in IADL impairment was associated with higher odds of PV (OR: 1.24, [95% CI 1.09-1.42]), ER visit (OR: 1.33, [95% CI 1.17-1.52]), and hospitalization (OR: 1.53, [95%CI 1.32-1.76]). These findings highlight disparities in healthcare utilization. Longitudinal studies are needed to strengthen causality between physical impairment, health insurance, and healthcare utilization.

